# Supplementary material for: Identification of photoperiod-induced specific miRNAs in the adrenal glands of Sunite sheep (Ovis aries)
Source: Front Vet Sci. 2022 Jul 22;9:888207. doi: 10.3389/fvets.2022.888207 (PMC9354845; doi:10.3389/fvets.2022.888207)
Supplement: Supplementary file 1 [file Data_Sheet_1.ZIP › Supplementary file/miRDeep2 file.zip!/miRDeep2 file/Novel_Structure/SP42A3/Novel_47.pdf]

Provisional ID	:	NC_019484.2_31607
Score total	:	13
Score for star read(s)	:	3.9
Score for read counts	:	5.2
Score for mfe	:	2.2
Score for randfold	:	1.6
Score for cons. seed	:	
Total read count	:	22
Mature read count	:	19
Loop read count	:	0
Star read count	:	3

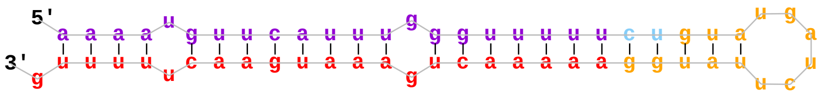

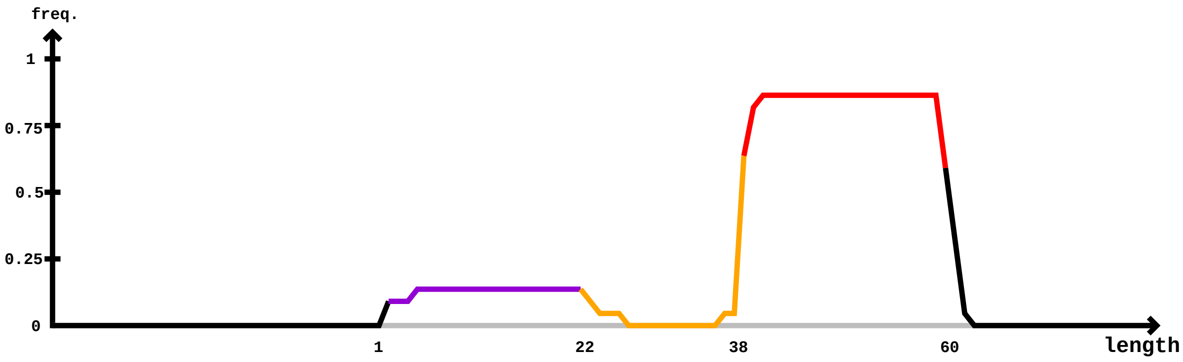

Star **Mature**

```
5'- guaaaaugaaaagucuucuguauuggguuggccaaaauguucauuuggguuuuucuguaugaucuuauggaaaaacugaaaugaacuuuuuggccaacucaauaucauacuca   -3'   obs
    guaaaaugaaaagucuucuguauuggguuggccaaaauguucauuuggguuuuucuguaugaucuuauggaaaaacugaaaugaacuuuuuggccaacucaauaucauacuca         exp
    ...................((((((((((((((((((.((((((((.((((((((((((......)))))))))))).)))))))).))))))))))))))))))........   reads   mm   sample
    .................................aaaaGguucauuuggguuuuu...........................................................   2       1    seq
    ....................................aAguucauuuggguuuuucugu.......................................................   1       1    seq
    ....................................................................ggaaaaacuCaaaugaacuuuuu......................   1       1    seq
    ......................................................................aaaaacugGaaugaacuuuuu......................   2       1    seq
    ......................................................................aaaaacuCaaaugaacuuuuu......................   3       1    seq
    ......................................................................aaaaacuCaaaugaacuuuuug.....................   6       1    seq
    ......................................................................aaaaacuCaaaugaacuuuuugg....................   1       1    seq
    ......................................................................aaaaacugaaaugaacuuuuugg....................   1       0    seq
    .......................................................................aaaacuCaaaugaacuuuuug.....................   4       1    seq
    ........................................................................aaacugGaaugaacuuuuuggc...................   1       1    seq
```
